# Efficacy and safety of artesunate–amodiaquine and artemether–lumefantrine and prevalence of molecular markers associated with resistance, Guinea: an open-label two-arm randomised controlled trial

**DOI:** 10.1186/s12936-020-03290-w

**Published:** 2020-06-24

**Authors:** Abdoul Habib Beavogui, Alioune Camara, Alexandre Delamou, Mamadou Saliou Diallo, Abdoulaye Doumbouya, Karifa Kourouma, Patrice Bouedouno, Timothée Guilavogui, Samaly dos Santos Souza, Julia Kelley, Eldin Talundzic, Aissata Fofana, Mateusz M. Plucinski

**Affiliations:** 1 Centre National de Formation et de Recherche en Santé Rurale de Maferinyah, Forécariah, Guinea; 2 Department of Medical Sciences, Gamal Abdel Nasser University, Conakry, Guinea; 3 Centre d’Excellence Africain pour la Prévention et le Contrôle des Maladies Transmissibles (CEA-PCMT), Gamal Abdel Nasser University, Conakry, Guinea; 4 Department of Public Health, Gamal Abdel Nasser University, Conakry, Guinea; 5 National Malaria Control Programme, Conakry, Guinea; 6 Direction of Epidemiology, Ministry of Health, Conakry, Guinea; 7 Malaria Branch, Centers for Disease Control and Prevention, Atlanta, USA; 8 Atlanta Research and Education Foundation and Malaria Branch, Centers for Disease Control and Prevention, Atlanta, USA; 9 RTI International, Conakry, Guinea; 10 Malaria Branch and U.S. President’s Malaria Initiative, Centers for Disease Control and Prevention, Atlanta, USA

**Keywords:** Efficacy, *Plasmodium falciparum*, Artesunate–amodiaquine, Artemether–lumefantrine, Molecular makers, Guinea

## Abstract

**Background:**

Anti-malarial resistance is a threat to recent gains in malaria control. This study aimed to assess the efficacy and safety of artesunate–amodiaquine (ASAQ) and artemether–lumefantrine (AL) in the management of uncomplicated malaria and to measure the prevalence of molecular markers of resistance of *Plasmodium falciparum* in sentinel sites in Maferinyah and Labé Health Districts in Guinea in 2016.

**Methods:**

This was a two-arm randomised controlled trial of the efficacy of AL and ASAQ among children aged 6–59 months with uncomplicated *Plasmodium falciparum* malaria in two sites. Children were followed for 28 days to assess clinical and parasitological response. The primary outcome was the Kaplan–Meier estimate of Day 28 (D28) efficacy after correction by microsatellite-genotyping. Pre-treatment (D0) and day of failure samples were assayed for molecular markers of resistance in the *pfk13* and *pfmdr1* genes.

**Results:**

A total of 421 participants were included with 211 participants in the Maferinyah site and 210 in Labé. No early treatment failure was observed in any study arms. However, 22 (5.3%) participants developed a late treatment failure (8 in the ASAQ arm and 14 in the AL arm), which were further classified as 2 recrudescences and 20 reinfections. The Kaplan–Meier estimate of the corrected efficacy at D28 was 100% for both AL and ASAQ in Maferinyah site and 99% (95% Confidence Interval: 97.2–100%) for ASAQ and 99% (97.1–100%) for AL in Labé. The majority of successfully analysed D0 (98%, 380/389) and all day of failure (100%, 22/22) samples were wild type for *pfk13*. All 9 observed *pfk13* mutations were polymorphisms not associated with artemisinin resistance. The NFD haplotype was the predominant haplotype in both D0 (197/362, 54%) and day of failure samples (11/18, 61%) successfully analysed for *pfmdr1*.

**Conclusion:**

This study observed high efficacy and safety of both ASAQ and AL in Guinea, providing evidence for their continued use to treat uncomplicated malaria. Continued monitoring of ACT efficacy and safety and molecular makers of resistance in Guinea is important to detect emergence of parasite resistance and to inform evidence-based malaria treatment policies.

## Background

Worldwide, malaria represents a constant and persistent public health threat due to its morbidity and mortality especially among children [1], with sub-Saharan Africa bearing the largest proportion of the burden [2]. Significant progress had been made in the control of malaria over the past years through interventions such as early case identification and diagnosis and prompt treatment with artemisinin-based combination therapy (ACT) [3]. Major artemisinin-based combinations used globally include artesunate–amodiaquine (ASAQ), artemether–lumefantrine (AL), and dihydroartemisinin–piperaquine (DP) [4].

However, progress in providing effective treatment of malaria is facing challenges including parasite resistance to anti-malarial drugs, particularly in South East Asia [5]. In response to these major threats, the World Health Organization (WHO) recommends periodic surveillance of anti-malarial first- and second-line treatment efficacy to provide data to national programmes for evidence-based malarial treatment policies [6]. This strategy is based on assessing both clinical and biological parameters along with analysis of molecular makers of resistance [4].

In line with these recommendations, sub-Saharan African countries have initiated the surveillance of the efficacy and safety of anti-malarial drug treatments to prevent parasite resistance [7–10]. In Tanzania, a study assessing the efficacy and safety of AL for the treatment of uncomplicated *Plasmodium falciparum* malaria and prevalence of artemisinin resistance molecular markers found high efficacy and safety of AL and no known artemisinin resistance *pfk13* mutations [11]. Several rounds of therapeutic efficacy monitoring in Angola have highlighted absence of molecular markers for artemisinin resistance along with generally high observed efficacies of ACT, albeit with some evidence of decreased AL efficacies [9, 12, 13]. In Gabon, a recent open-label clinical trial reported lack of molecular markers of artemisinin resistance and high efficacy of artemether–lumefantrine and artesunate–amodiaquine [14]. A review and network meta-analysis monitoring the efficacy and safety of ACT in Cameroon, reported that ACT is still effective and safe in Cameroon, but there are insufficient data on efficacy, safety and tolerability [15]. In Guinea, initial studies prior to ACT introduction showed high baseline efficacy [16, 17]. More recent data from a large multi-site randomised control trial have showed continued efficacy of a range of artemisinin-based combinations in Guinea and the larger West Africa region [2].

In Guinea, ACT has been recommended as a first-line anti-malarial treatment for uncomplicated *Plasmodium falciparum* infection since 2005, with both ASAQ and AL included in the national treatment guidelines. Prior to 2016, ASAQ was the primary artemisinin-based combination used in Guinea, but since then AL has largely replaced ASAQ. The change in ACT medicine procurement strategy was motivated by patient and provider preference [18] as well as the expansion of seasonal chemoprevention using sulfadoxine–pyrimethamine and amodiaquine [19].

Guinea has observed a significant decrease of malaria burden over the past years, with malaria prevalence in children < 5 years measured in the last national household surveys declining from 44% in 2012 to 30% in 2017 [20]. However, in light of limited data on resistance molecular makers in the region, in 2015 the National Malaria Control Programme (NMCP) began implementing periodic therapeutic efficacy studies rotating between four sentinel sites in the country for early detection of emergence and prevention of spread of drug resistance.

The present study aimed to assess the efficacy and safety of ASAQ and AL in the management of uncomplicated malaria in children aged 6–59 months and to measure the prevalence of molecular markers of resistance of *Plasmodium falciparum* in two sentinel sites in Guinea in 2016.

## Methods

### Study sites

This study was conducted in two of the four sentinel sites across the four natural regions of the country: Maferinyah health centre in Forécariah District in Lower-Guinea and Ley-Sare health centre in Labé District in Middle-Guinea. Anti-malarial resistance surveillance in Guinea rotates between four sentinel sites and the 2016 round occurred in Maferinyah and Labé. Maferinyah is a hyperendemic area with high rainfall (6 to 10 months), and the principal vector of malaria is *Anopheles gambiaesensu stricto* (*s.s*.). Labé is a mesoendemic area with low rainfall, with *Anopheles funestus* and *Anopheles melas* as primary vectors [21].

### Study design, period and population

This was an open-label two-arm randomised controlled trial assessing the therapeutic efficacy of two anti-malarial treatments: ASAQ and AL among children aged 6 to 59 months with uncomplicated *Plasmodium falciparum* malaria. Participants meeting study inclusion criteria were allocated to either group in a 1:1 ratio, stratified by study site. The allocation was restricted with randomly varying block sizes of 4–6 and was concealed through sealed opaque white envelopes.

### Sample size

A non-probability sampling methodology was used to select patients presenting at the two study sites. A minimum of 100 patients per treatment arm were required giving a total of 200 children per site. The sample size was determined based on a precision of ± 5% for the proportion of clinical and parasitological cure at Day 28 (D28) after correction by PCR assuming a cure rate of 95% and a loss to follow-up of 25%.

### Inclusion criteria

Patients were screened and included according to the WHO standard protocol related to the treatment of uncomplicated *Plasmodium falciparum* malaria (2009) [22]. Briefly, children aged 6–59 months, inclusive, with axillary temperature ≥ 37.5 °C or history of fever in last 24 h and microscopy-confirmed *Plasmodium falciparum* monoinfection with parasitaemia between 2000 and 200,000 p/μl without signs of severe malaria and available for the full period of follow up were invited to participate.

### Treatment

Patients meeting the inclusion criteria were treated with either ASAQ or AL based on randomisation on site by an authorised member of the research team and they were given the drug according to their weight and age. The treatment duration was 3 consecutive days (D0, D1 and D2) with oral intake under medical observation once daily for ASAQ and twice daily for AL. The entire dose was repeated if vomiting occurred within 30 min after intake of the anti-malarial and half the dose if vomiting occurred between the first 30 min and 1 h after intake. Drugs such as vitamin C [23], vitamin B12 [24], retinol supplementation [25, 26], that may influence anti-malarial drug activity were avoided during prescription of concomitant drugs. Similarly, antibiotics such as cotrimoxazole, macrolides, tetracycline and doxycycline were avoided during the period of follow up.

### Clinical follow-up

Patients were actively followed for 28 days for both arms from the 1st day of treatment D0 till D28 with scheduled visits on D1, D2, D3, D7, D14, D21 and other days of unscheduled visits. Patients were systematically assessed on D0 for splenomegaly according to the Hackett classification that has been used in previous studies [27, 28]. Clinical and parasitological assessments were performed and dried blood spots were collected on all days of follow-up with the following exceptions: microscopy was only performed on D1 in the presence of signs of severe malaria and no dried blood spots were collected on D1–D2. Recurrent infections were assessed by the field medical doctor of the site and treated with quinine (injection/intravenous) or artesunate injection based on clinical presentation as per the Guinean National Malaria Control Programme protocol.

The on-site research team was available to ensure 24-h passive monitoring for patients. During enrollment and scheduled visits, parents/guardians were informed and encouraged to bring back their children to the health centres or call the field medical doctor whenever their children felt unwell without waiting for scheduled visits. Patients who did not show up for their scheduled visits by mid-day were first called and asked to come to the health centre and then actively searched for by a community health worker. If a patient had travelled and could not be traced for scheduled follow-up, he or she was classified as lost to follow-up.

### Data collection

Data were collected using a standardised case report form. Data variables included sociodemographic characteristics (age, gender), clinical characteristics (weight, body temperature, splenomegaly) and laboratory results (malaria microscopy and haemoglobin).

### Laboratory monitoring

#### Parasitaemia and haemoglobin

Malaria microscopy was carried out using 10% Giemsa staining of thick and thin smears according to a standard operating procedure on D0 and every day of follow-up except D1. The parasite density from the thick smear was determined according to the following formula: Parasitaemia per microlitre = (number of asexual parasites divided by 200 or 500 counted leukocytes) multiplied by 8000. A slide was classified negative when the entire examination of the thick smear revealed no asexual form of *Plasmodium*. The presence of gametocytes of *Plasmodium falciparum* (sexual forms) was determined over 1000 leucocytes instead of 200 or 500 for the asexual forms. For quality control, each slide was read by two microscopists and, if results differed by more than 30%, were re-examined by a third microscopist, with the final parasite density calculated based on the two closest results. Haemoglobin was measured using HemoCue machines (AB Leo Diagnostics, Helsinborg, Sweden).

#### Biomolecular markers

Dried blood spots were collected on Whatman 903 filter paper on D0, D3, D7, D14, D21, D28 and at any unscheduled visits. Fragment lengths of seven neutral microsatellite markers (Additional file 1: Table S1) were used to compare genotypes on D0 and day of failure for patients with recurrent parasitaemia using a Bayesian classifier for molecular correction [29]. In brief, the Bayesian algorithm uses allele frequencies to calculate the posterior probability of recrudescence for each recurrent parasitaemia. Patients with a posterior probability of recrudescence greater than 0.5 were considered as recrudescences in the analysis.

Additionally, all D0 samples and Day of Failure samples from late treatment failures were systematically amplified and sequenced for *pfk13* and *pfmdr1* resistance genes following previously described methodologies [30]. Molecular analyses were performed in collaboration with the U.S. Centers for Disease Control and Prevention (CDC) laboratories in Atlanta, USA as part of the PMI-supported Anti-malarial Resistance Monitoring in Africa (PARMA) Network [31].

### Study outcomes

The primary endpoints were adequate clinical and parasitological response (ACPR), early treatment failure (ETF), and late treatment failure (LTF) in accordance with the WHO in vivo guidelines. Secondary endpoints of therapeutic efficacy included the proportion of patients with negative slides at D3. Adverse events that occurred were reported on specific forms and classified according to their severity and their assessed relationship to the study. Serious or unexpected side effects were reported to the principal investigator, the sponsor, the study coordinator and the Guinean Ethics Committee on Health Research.

### Data analysis

Data from the standardised forms were double-entered into Microsoft Access version 2010 (Microsoft Corporation, Redmond, WA) and then exported into STATA 14 software (Stata Corporation, College Station, TX, USA) for analysis. Primary endpoints findings were tabulated, and the primary outcome was reported as the Kaplan–Meier estimate of the corrected efficacy at D28 by study site and drug.

## Results

### Baseline characteristics of participants enrolled and completing follow-up

A total of 966 participants were screened from July to October 2016 at the two study sites. A total of 211 and 210 participants were included in the Maferinyah and Labé sites, respectively, for a total of 421 participants. Baseline characteristics of the included participants who completed their follow-up are shown in Table 1. At both study sites, nearly all included participants completed their follow-up, with combined exclusion and loss to follow-up rates of less than 3% across all arms. The median age of the study participants was 36 months [interquartile range (IQR): 24–48] for both study arms in Maferinyah. In Labé, median patient age was higher in the ASAQ arm [45 months, (IQR): 24–59] than in the AL arm [36 months, (IQR): 24–48]. The median weight of the participants at both sites was similar across all arms, at approximately 13 kg.

**Table 1:** Baseline characteristics of participants enrolled and completing follow-up (n = 421)

Variables	Maferinyah (n = 211)		Labé (n = 210)	
	ASAQ	AL	ASAQ	AL
Participant characteristics at baseline				
Median age, in month (interquartile range)	36 (24–48)	36 (24–59)	45 (24–59)	36 (24–48)
Median weight, kg (interquartile range)	14 (10–16)	13 (11–16)	13 (10–16)	13 (10–15)
Percent female, n (%)	55 (51.4)	39 (37.5)	40 (38.1)	43 (41.0)
Median day 0 parasitemia, parasites/µl * 10^3^ (interquartile range)	19.4 (101–519)	27.4 (10.2–55.0)	30.2 (13.0–63.9)	30,450 (13.1–49.6)
Median day 0 haemoglobin, g/dl (interquartile range)	9.7 (8–10)	9.8 (8–10)	10.7 (9–11)	10.6 (8.5–11)

*AL* artemether–lumefantrine, *ASAQ* artesunate–amodiaquine

Overall, there were fewer girls than boys among the included participants at both sites (39.5% vs 60.5% in Labé and 44.5% vs 55.5% in Maferinyah). At inclusion (D0), the median baseline parasite density for participants in AL arm was higher at 19,400 parasites/μl [(IQR): 10,100–51,900] than for those in ASAQ arm 27,430 parasites/μl [(IQR): 10,215–55,045] in Maferinyah. In Labé, median baseline parasite density was almost the same in both arms at 30,200 and 30,480 parasites/μl respectively. The median haemoglobin level of participants, was 9 g/dl for both arms in Maferinyah 10 g/dl for both arms in Labé.

### Rates of follow up and treatment outcomes of participants who completed their follow-up

Of the 421 included participants, 8 (1.9%) were lost to follow-up, including two deaths. This left 413 participants completing their follow-up. Treatment outcomes of participants who completed their follow-up are shown in Table 2. No ETF was observed in any study arms. In contrast, 22/413 (5.3%) participants developed a late treatment failure: 5/105 (4.8%) in the Maferinyah ASAQ arm, 8/101 (7.9%) in the Maferinyah AL arm, 3/104 (2.8%) in the Labé ASAQ arm and 6/103 (5.8%) in Labé AL arm.

**Table 2:** Treatment outcomes for participants finishing follow-up as part of therapeutic efficacy monitoring in Guinea, 2016 (N = 413)

	Maferinyah		Labé	
	ASAQ	AL	ASAQ	AL
Enrolled	107	104	105	105
Reached study outcome	105	101	104	103
Treatment failure	5 (5)	8 (8)	3 (3)	6 (6)
Early treatment failure	0	0	0	0
Late treatment failure	5 (5)	8 (8)	3 (3)	6 (6)
Recrudescence	0	0	1 (1)	1 (1)
Day 28	0	0	1 (1)	1 (1)
Reinfection	5 (5)	8 (8)	2 (2)	5 (5)
Day 21	1 (1)	3 (3)	1 (1)	1 (1)
Day 28	4 (4)	5 (5)	1 (1)	4 (4)
Adequate clinical and parasitological response	100 (95)	93 (92)	101 (97)	97 (94)
Kaplan–Meier Day 28 efficacy				
Uncorrected	95.2% (91.3–99.4)	92.1% (87.1–97.5)	97.1% (94–100)	94.2% (89.9–98.8)
Microsatellite-corrected	100%^a^	100%^a^	99% (97.2–100)	99% (97.1–100)

*AL* artemether–lumefantrine, *ASAQ* artesunate–amodiaquine, 28-day follow-up

^a^confidence intervals: undefined

Of the 22 late treatment failures, 2 were classified as recrudescences and 20 were classified as reinfections (Additional file 1: Table S1). Both recrudescences occurred at D28 of follow-up, one in the Labé AL arm and one in the Labé ASAQ arm. The Kaplan–Meier estimate of the D28 corrected efficacy rate was 100% (CI 100–100%) for the Maferinyah AL arm, 100% (CI 100–100%) for Maferinyah ASAQ arm, 99% (CI 97.1–100%) for the Labé AL arm, and 99% (CI 97.2–100%) for Labé ASAQ arm.

### Safety of the anti-malarial drugs and deaths observed

Vomiting at any time during treatment was observed in 59/421 (14.0%) participants with 26 (44.1%) in the AL arm and 33 (53.9%) in the ASAQ arm. Two participants (one in the Maferinyah ASAQ arm and one in the Maferinyah AL arm) developed signs of severe malaria less than 24 h after inclusion in the study; one (in the Maferinyah AL arm) ultimately died. Both were excluded from analysis due to onset of severe symptoms less than 24 h after inclusion following WHO definitions. One additional participant in the Maferinyah ASAQ arm died from a car accident during follow-up, and was censored at day 14 in the analysis.

### Results of Day 2 and Day 3 microscopy

The proportion of negative slides at D2 and D3 of follow-up is shown in Table 3. At D2 of follow-up, the vast majority of the participants had negative slides at examination at both sites and arms (> 88% for the two arms in Maferinyah and > 90% in Labé). At D3 of follow-up, nearly all slides were negative at both sites and in both arms (> 99% in Maferinyah and 100% in Labé).

**Table 3:** Proportion of slides negative for asexual malaria parasites on days 2 and 3 following treatment (n = 413)

Variables	Maferinyah (n = 206)				Labé (n = 207)			
	ASAQ	%	AL	%	ASAQ	%	AL	%
Day 2	94	89.5	89	88.1	99	95.2	94	91.3
Day 3	103	99.0	102	100	103	100	100	100

### Molecular markers of resistance

DNA was isolated from 443 samples, including 421 pretreatment and 22 day-of-late treatment failure samples. Of the 443 samples analysed, 411 (93%) were successfully amplified and sequenced for *pfk13* (Table 4). The majority of D0 (98%, 380/389) and all day-of-late treatment failure (100%, 22/22) samples were wild type for *pfk13*. All 9 observed *pfk13* mutations were polymorphisms that have not been associated with artemisinin resistance.

**Table 4:** Prevalence of molecular markers of resistance in Day 0 and Day of Failure samples from therapeutic efficacy studies in Guinea, 2016

	Day 0	Day of Failure
*pfk13*	n = 389	n = 22
Wild-type	380 (98%)	22 (100%)
Mutant	9 (1%)^a^	0 (0%)

*pfmdr1* 86 codon	n = 379	n = 18
N	296 (78%)	14 (78%)
Y	59 (16%)	4 (22%)
N/Y	24 (6%)	0 (0%)

*pfmdr1* 184 codon	n = 379	n = 18
Y	117 (31%)	3 (17%)
F	206 (54%)	14 (78%)
Y/F	56 (15%)	1 (6%)

*pfmdr1* 1246 codon	n = 370	n = 18
D	363 (98%)	18 (100%)
Y	6 (2%)	0 (0%)
D/Y	1 (0.3%)	0 (0%)

*pfmdr1* haplotypes^b^	n = 362	n = 18
NYD	158 (44%)	4 (22%)
YYD	17 (5%)	0 (0%)
NFD	197 (54%)	11 (61%)
YFD	71 (20%)	4 (22%)
NYY	1 (0.3%)	0 (0%)
YYY	6 (2%)	0 (0%)
NFY	1 (0.3%)	0 (0%)
YFY	0 (0%)	0 (0%)

^a^All 9 samples with polymorphisms not associated with artemisinin resistance: P419S (1), L429L (1), C469C (3), G496G (1), E509E (1), V510V (1), A621A (1)

^b^Haplotypes defined at codons 86, 184, and 1246. Samples with multiple haplotypes were included in the numerator for each haplotype

Amplification and sequencing of the *pfmdr1* gene at the 86, 184, and 1246 codons was successful in 380/443 samples (86%). The NFD haplotype was the predominant *pfmdr1* haplotype at D0, present in 54% (197/362) of analysable D0 samples, followed by the NYD haplotype, present in 44% (158/362) of D0 samples. In late treatment failure samples, the NFD haplotype also predominated, at 61% (11/18).

## Discussion

This study marks the first round of anti-malarial resistance monitoring in Guinea, which has to date lacked regular and systematic surveillance since the introduction of ACT. The results showed high efficacy of ASAQ and AL (microsatellite-corrected D28 efficacies > 99%) to treat uncomplicated malaria, despite their use for more than a decade in Guinea. These results are consistent with the high ACT efficacies observed prior to introduction of ACT in Guinea and with other studies from Africa [8, 11, 32, 33].

Rates of recurrent parasite density were relatively low in the Guinea study sites, below 8% in the 28-day follow up period. This low rate of recurrent parasite density may be explained by a high proportion of children < 5 years sleeping under an insecticide-treated net in Guinea as reported by the last demographic and health survey (2018) of the country [34]. The results showed a high rate of parasite clearance on D3 of follow-up at both sites and in both arms, with all patients slide negative by D3. This finding is similar to other TES studies conducted throughout the continent [35] but contrasts with the slow parasite clearance rate of AL in the Greater Mekong region [5, 36]. The high rate of parasite clearance with AL and ASAQ found in this study indicates high susceptibility of the parasite to the artemisinin component of the combination, which is able to rapidly reduce parasite biomass [37, 38]. Absence of *pfk13* mutations associated with artemisinin resistance is further evidence that parasites remain susceptible to artemisinin derivatives in Guinea. This finding is similar to those studies in Africa [11, 30, 39–42] reporting a lack of the *pfk13* mutations associated with artemisinin combination therapeutic resistance that have been identified in Southeast Asia [40, 43–45].

Sequencing of the *pfmdr1* gene revealed that the majority of both pre-treatment samples and late treatment samples harbored the NFD haplotype, a molecular maker associated with reduced susceptibility of *Plasmodium falciparum* to lumefantrine [46, 47]. As AL use increases in Guinea following the treatment policy switch to AL, continued surveillance of the prevalence of NFD *pfmdr1* haplotype is important.

The current study only reports efficacy data from two sites in the country, and the results may not be representative of the whole country. Subsequent therapeutic efficacy studies from the remaining two sentinel sites in N’Zérékoré and Dabola health districts will further inform monitoring of anti-malarial resistance.

## Conclusion

This study found high efficacy and safety of ACT in Guinea, providing evidence that supports continued use of ASAQ and AL to treat uncomplicated malaria. Continued monitoring of ACT efficacy and safety and molecular makers of resistance in Guinea are important to detect parasite resistance and to inform evidence-based malaria treatment policies.

## Supplementary information

**Additional file 1: Table S1.** Observed fragment lengths of neutral microsatellite loci from paired Day 0 (D0) and Day of Failure (DOF) samples from therapeutic efficacy studies in Guinea, 2016.

## Data Availability

All data generated or analysed during this study are available from the corresponding author on reasonable request.

## Abbreviations

ACPR: Adequate clinical and parasitological response
ACT: Artemisinin-based combination therapy
AL: Artemether–lumefantrine
ASAQ: Artesunate + amodiaquine
CDC: Centers for Disease Control and Prevention
CI: Confidence interval
CNERS: Comité National d’Ethique pour la Recherche en Santé
DNA: Deoxyribonucleic acid
ETF: Early treatment failure
IQR: Interquartile range
LPF: Late parasitological failure
LTF: Late treatment failure
NMCP: National Malaria Control Programme
PARMA: PMI-supported antimalarial resistance monitoring in Africa
PCR: Polymerase chain reaction
*pfk13*: *Plasmodium falciparum* Kelch 13 gene
*pfmdr1*: *Plasmodium falciparum* multi drug resistance gene 1
PMI: President’s Malaria Initiative
TES: Therapeutic efficacy study
USA: United States of America
WHO: The World Health Organization
