# Supplementary material for: Efficacy and safety of artesunate–amodiaquine and artemether–lumefantrine and prevalence of molecular markers associated with resistance, Guinea: an open-label two-arm randomised controlled trial
Source: Malar J. 2020 Jun 24;19:223. doi: 10.1186/s12936-020-03290-w (PMC7315541; doi:10.1186/s12936-020-03290-w)
Supplement: Supplementary file 1 — Additional file 1: Table S1. Observed fragment lengths of neutral microsatellite loci from paired Day 0 (D0) and Day of Failure (DOF) samples from therapeutic efficacy studies in Guinea, 2016. [file 12936_2020_3290_MOESM1_ESM.pdf]

Additional file 1: Observed fragment lengths of neutral microsatellite loci from paired Day 0 (D0) and Day of Failure (DOF) samples from therapeutic efficacy studies in Guinea, 2016.

Chromosome	6				6		106		4			2			3		12				
Tandem Reapets	TAA				ATT		TAA			ATT		AT			TA		TAA				
Marker Name	TA109				TA1		2490		POLYA			313			383		PFPK2				
Sample ID	TA109_1	TA109_2	TA109_3	TA109_4	TA1_1	TA1_2	2490_1	2490_2	POLYA_1	POLYA_2	POLYA_3	313_1	313_2	313_3	383_1	383_2	PFPK2_1	PFPK2_2	PFPK2_3	Probability of Recrudescence	Final Classification
01-017_ D0	164	175			170		89		151			255			271		156			0,02	Reinfection
01-017_DOF	164	175			168		82		180			146	236		137		168				
01-020_ D0	149	160			159		x		142	154		x			124		169			0,01	Reinfection
01-020_DOF	179	160			169		82		165			245			144		174				
01-095_ D0	160	163	197		169	202	78	82	152	164		239			126	145	177	174		0,01	Reinfection
01-095_DOF	151	160			171		82		135			244			141		172	173	180		
01-096_ D0	160	173			166		78	82	142	155	168	230	243	245	124		161	159		0,03	Reinfection
01-096_DOF	160	174	188		153		82		144			203	228		143		166				
01-108_ D0	179	197			178		81		180			226			123		161			0,00	Reinfection
01-108_DOF	149	160			170		81		151			257			141		159				
01-113_ D0	163	176			175	187	82	88	168	177		222	245		128	145	161			0,00	Reinfection
01-113_DOF	175				160		18	82	157	177		140	226		125	152	162	180			
01-120_ D0	163	176	199		160		82		131			243			124		159			0,02	Reinfection
01-120_DOF	164	175			166	175	82		151	162		234			124		161				
01-123_ D0	165	175			175	162	82	88	167	177		245			128		162	165		0,03	Reinfection
01-123_DOF	165	175			160		81		157	176		225			151		180				
01-124_ D0	164	170			167		81	88	154			215	251		124		154	159		0,00	Reinfection
01-124_DOF	149	159			181		78		152			238			152		165				
01-168_ D0	162	172			178		82		150			232			145		161			0,00	Reinfection
01-168_DOF	150	159			163	190	82		154			261			142		176	186			
01-175_ D0	149	160			186		82		154			240			150		165			0,00	Reinfection
01-175_DOF	151	163	175		159	169	79	82	152	171		226	237		113		161	169			
01-191_ D0	185				165		82		157			140	236		122		166			0,02	Reinfection
01-191_DOF	150	159	162		169		81		151	157		249			159		165	175			
01-197_ D0	150	159	175		164	172	82	85	154	151		230			123		168			0,00	Reinfection
01-197_DOF	156	167			175		82		142			217			143		159				
02-012_ D0	164	176			169		82		148			228	239		135		174			0,00	Reinfection
02-012_DOF	196				74		x		x			140			x		x				
02-014_ D0	161	173			173		83	88	134			239	255		142		165			0,00	Reinfection
02-014_DOF	136	147			142		72		160			140	243		146		165				
02-019_ D0	161	164			168	183	82		174			240	262		123		159	162		0,00	Reinfection
02-019_DOF	173	178			174		79		152			264			139		176				
02-036_ D0	175	184			171	186	82	79	149	158		234	240		130	147	168			0,00	Reinfection
02-036_DOF	161	173			175		81	78	161			253			147		161				
02-055_ D0	148	160			130		82		151			253			139		176			0,93	Recrudescence
02-055_DOF	149	161			130		82		152			140	253		139		177				
02-065_ D0	158	169			159		81		151			234			138		161			0,04	Reinfection
02-065_DOF	160	168	173		172	175	82	78	145	149		230			140		161	173	180		
02-074_ D0	177	187			169		72		161			73	140	238	123		162			0,06	Reinfection
02-074_DOF	162	192	184		168		82		148	161		247			139		168				
02-088_ D0	150	160			178		78		137			247			148		183			0,02	Reinfection
02-088_DOF	161	164			74	172	x		149	165		221	230	245	x		165				
02-133_ D0	164	175	178		163	175	82	88	158	168	177	226			123	128	165	186		0,69	Recrudescence
02-133_DOF	64	175	178	179	159		79	82	158			226			151		161	180			
